# Muscular Rheumatism, as Connected with Atmospheric Electricity

**Published:** 1849-08

**Authors:** N. B. Sherwood

**Affiliations:** Conneautville, Pa.


ART. II.—*Muscular Rheumatism, as connected with Atmospheric Electricity.* By N. B. Sherwood, M. D.

Mr. Editor:—Allow me to lay before you the following remarks on a form of rheumatism which occurs very frequently, and which is influenced, in a remarkable degree, by *approaching* transitions of weather. The disease to which I refer is usually styled, very properly, "Muscular Rheumatism." It is very aptly described by Macleod in his work on rheumatism. He says, if I mistake not, "that it differs from the sequela of acute rheumatism," and further, that it "consists, in the milder cases, of a dull uneasiness in the part while at rest, which, however, becomes converted into a sharp cutting pain when it is moved. Sometimes, indeed frequently, there fort except on calling certain muscles into action, and if this from forgetfulness, or other cause, be done abruptly, the suffering is often so acute as to make the patient cry out involuntarily." Lumbago is very commonly a concomitant of muscular rheumatism, or rather it is a very characteristic form of it. The disease may attack any muscle, or set of muscles, but is most frequently seated in those of the hips, back, and intercostal space. It is, like other forms of rheumatism, more acute in the night than in the day. Its "obstinacy is proverbial, and is much aggravated by atmospherical vicissitudes," says Johnson, and many other authors, in speaking of it, have attributed it entirely to atmospherical changes. This disease is one of interest to me, from the fact that I am severely afflicted by it, having been rheumatic some five or six years, during which time I have carefully made many observations, and have found that transitions of weather do indeed aggravate the disease, but, in addition, have found that something more than a mere transition is necessary to produce the effect.

There is nothing strange in the simple fact that a disease may be modified by a particular condition of the atmosphere, but it is strange that such modification does, in a great many instances, take place while yet the atmosphere surrounding the patient remains unchanged so far as common observation and barometrical indications are regarded.

It has been observed for a long time, that some rheumatic patients have the faculty of foretelling a storm, the only barometer consulted being their own pains and aches. To the solving of the question,—how does the storm act on the patient at such distances as it sometimes does—I have applied myself. Of a bright sunny day you may hear a patient foretelling a storm. No indications are to be observed, the barometer is fair, the air balmy and warm, and none save the rheumatic are aware of the coming storm; how are we to solve this? It certainly cannot be attributed to a change in the density of the surrounding atmosphere, for here the barometer would be acted upon. My observations, extended through a period of five years, with myself for the patient, have led me to the conclusion that it may be solved by the application of the theory of electrical disturbances, and further, that these disturbances are produced by the rotation of storms.

I would state here, however, that a simple rain storm, unattended by wind, rarely affects me so much as one consisting entirely of wind, and the last not so much as one attended by both rain and wind.

To prove the correctness of my theory, I fear, will prove a difficult task, but I attempt it by citing the experiments conducted by a gentleman whose name I have forgotten. He applied to the human subject an extensive coating of varnish composed of shellac; the patient had been troubled with rheumatism, the shellac coating *aggravated the disease,* while a coating of tin foil *lessened the disease.* Whether this statement can be good evidence, I know not. It is not material to my purpose. I cite it merely as a statement.

Again, it has been proved by repeated experiments that the patient, while in the most painful condition, is essentially in a *negative state.* My own experiments on myself satisfy me that such is the case. I shall not attempt to connect the two foregoing statements, or to explain the *modus operandi* of the coatings. I cite the two to show that "a disturbance in the terrestrial electrical currents produces symptoms analagous to those produced by storms.

The question now is, how are these disturbances effected? I answer by rotation.

We must now turn our attention to facts proclaimed by M. Arago, and confirmed and explained by Faraday. "If a circular plate of copper be made to revolve immediately above or below a magnetic needle or magnet, suspended in such a manner that the magnet may rotate in a plane parallel to that of the copper plate, the magnet tends to follow the circumvolution of the plate; or if the magnet revolves, the plate tends to follow its motion." * * "This is quite independent of the motion of the air, as a glass plate interposed did not affect the result." Arago states that this takes place not only with metals, but with all substances, solids, liquids and even gases.

Mr. Faraday, however, did not stop here. He showed that "if a disc of copper be made to revolve in a plane passing through the line of the dip, no electrical phenomena are developed, but if the plane of revolution be changed to any other, at any angle to the first plane, all the phenomena of the plates with the magnets are shown, and the intensity of the electrical disturbance is exactly as the acuteness is less, and arrives at the maximum when the plate revolves at right angles to the line of the dip. When the revolution is in the same direction with the hands of a watch the current of electricity flows from the centre to the circumference, and when the rotation is in a contrary direction, the current sets directly to the reverse."

With these facts we must now couple others. It has been very conclusively proved that storms are rotary, having an axis of rotation, that this axis is in a great many instances at a very acute angle, with the line of the dip the plane of rotation, not coinciding, therefore, with the plane of the magnetic currents from the equator to the poles.

Here, then, we have one of Faraday's plate on a scale of surpassing magnitude, the periphery being either positive or negative, as the motion is from left to right, or the reverse. A simple fact may be cited to sustain this. Dr. L. C. Beck has in his possession a pane of glass with a perforation in the centre, almost mathematically circular. The perforation was caused by a hurricane which passed over New Brunswick some 16 years ago; this pane was found in this condition after the hurricane had passed. The wonder of the thing is, that the perimeter is smooth, presenting a fused appearance.

The only explanation is, that an enormous accumulation of electricity at the axis of rotation was obstructed in its descent to the earth by this pane of glass, and that the pole of the axis in rotation came in contact with the glass at this point, fusing the glass, and forcing a passage.

The distance, too, at which a storm acts sometimes on the rheumatic patient is worthy of notice, and can only be explained on the hypothesis of electricity. Let me, however, bring in some other facts worthy of attention. Allow me to state what have been the effects of a known rotation on a small scale.

I have been at various times engaged in practical mechanics, both in the shop and draughting room, and have noted, in a few cases, some very curious effects. The first shop was in the northern part of Illinois, the time, summer-weather, very pleasant, remarkably dry and salubrious; but notwithstanding all these various favorable circumstances, I was compelled to leave it on account of the most severe muscular rheumatism, but I did not leave the place for some days thereafter. My rheumatic pains left me within a few days, and occurred again when I again entered the shop under the impression that I was well enough to attend to my duties.

Now, if we reflect that the most simple movement of a body cannot take place without a disturbance of electrical currents, we may well believe the disturbance to be enormous in a machine shop, where so many complicated motions are in operation at the same time.

And this is the true cause of the many symptoms I felt in the shop I have mentioned, for on an investigation of the causes, I found the main shafts all running parallel to the line of the dip. Consequently the planes of revolution were at right angles to that line.

I could not be mistaken, for, as I said before, the weather was remarkably fine and healthy, and, what is more, the pains disappeared after leaving the work shop, and reappeared on again entering.

New let me present a case as well marked as the other. I was engaged for six weeks in a shop in the southern part of Ohio, in 1847. Like fall weather in general, we had sunshine and rain in rather uncomfortable proportions. The climate of that section of country is proverbially unsettled, and favorable to the development of rheumatism; the situation of the building, too, was highly prejudicial to health, being exposed to almost every wind, and the building itself very imperfectly enclosed. With all these disadvantages, however, I experienced less pain than I have in other situations unconnected with machine shops, and equally exposed. In the same section of country, in another shop, the state of the weather being nearly the same, and with but a few days intermission from shop to shop, I was most sorely afflicted, although the situation and general healthiness of the last were much better than the first. It may have been merely a coincidence, but the main shafts in the first shops in Ohio were all at right angles to the line of the dip, the reverse being the case in the second.

There was one circumstance connected with the last shop mentioned that impressed me strongly. My duties required me to frequently inspect a number of small rods which a workman was polishing on a large grind stone that revolved with considerable velocity. I invariably experienced very disagreeable sensations whenever I touched a rod that was in contact with the stone, and it was sometime before these sensations would leave me.

Now all these things may have been merely coincidences, colored by my hobbv, but, sir, it is not the less true that in every shop the angle of inclination and velocity of motion have borne a certain proportion to my rheumatic symptoms. The electricity of belts has long been familiar to those engaged in machinery; indeed, any one may satisfy himself by paying a very slight attention to a belt while in motion. It cannot be affirmed that the slipping of the bands on the drums occasion the current, for if such was in reality the case, every belt of equal tension, velocity and dimensions, should, and would exhibit the same results, but this is by no means the case.

The analogy of the storms and wheels can now be plainly seen. I have given my views, and, as imperfect as have been my statements, I am inclined to believe that you can gather from them all of my meaning.

*Conneautville, Pa.*

